# NO_2_ Physical-to-Chemical Adsorption Transition on Janus WSSe Monolayers Realized by Defect Introduction

**DOI:** 10.3390/molecules28041644

**Published:** 2023-02-08

**Authors:** Lin Ju, Xiao Tang, Xiaoxi Li, Bodian Liu, Xiaoya Qiao, Zhi Wang, Huabing Yin

**Affiliations:** 1 School of Physics and Electric Engineering, Anyang Normal University, Anyang 455000, China; 2 College of Science, Institute of Materials Physics and Chemistry, Nanjing Forestry University, Nanjing 210037, China; 3 Joint Center for Theoretical Physics, Institute for Computational Materials Science, School of Physics and Electronics, Henan University, Kaifeng 475004, China

**Keywords:** gas sensing, WSSe monolayer, Se vacancy, density functional theory

## Abstract

As is well known, NO_2_ adsorption plays an important role in gas sensing and treatment because it expands the residence time of compounds to be treated in plasma–catalyst combination. In this work, the adsorption behaviors and mechanism of NO_2_ over pristine and Se-vacancy defect-engineered WSSe monolayers have been systematically investigated using density functional theory (DFT). The adsorption energy calculation reveals that introducing Se vacancy acould result in a physical-to-chemical adsorption transition for the system. The Se vacancy, the most possible point defect, could work as the optimum adsorption site, and it dramatically raises the transferred-electron quantities at the interface, creating an obviously electronic orbital hybridization between the adsorbate and substrate and greatly improving the chemical activity and sensing sensitivity of the WSSe monolayer. The physical-to-chemical adsorption transition could meet different acquirements of gas collection and gas treatment. Our work broadens the application filed of the Janus WSSe as NO_2_-gas-sensitive materials. In addition, it is found that both keeping the S-rich synthetic environments and applying compression strain could make the introduction of Se vacancy easier, which provides a promising path for industrial synthesis of Janus WSSe monolayer with Se vacancy.

## 1. Introduction

With the advancement of science and technology, atmospheric pollution is increasingly worsening, thus causing continuous concern. NO_x_ are major hazardous air pollutants, of which NO_2_ is a threat to both the environment and human activities. With respect to human health, NO_2_ at concentrations greater than 1 ppm can seriously damages human lung tissue and the respiratory system, triggering or aggravating respiratory diseases, such as emphysema and bronchitis [1,2]. NO_2_ is able to generate acid rain, acid fog, and photochemical smog; as ecologically similar to CO_2_, it can also cause global warming. In spite of all these drawbacks, NO_2_ has some vital industrial applications, e.g., it can be used as a nitric acid component, a rocket propellant [3], a fungicide [4], and a disinfectant [5]. Owing to the above reasons, the detection, collection, and treatment of NO_2_ gas are considered to be extremely essential and have received increasingly close attention [6,7,8,9]. Numerous absorbent materials have been developed to absorb NO_2_, including activated carbon [10], metal–organics [11], metal oxide particles [12,13,14], etc. For example, bulk phase TiO_2_ has been described to be very efficacious as an adsorbent or catalyst in capturing and/or reforming NO_2_ [13,14], wherein NO_2_ gas molecules can react with metal centers, i.e., Ti^4+^ sites, via O, N, or a mixture of both. In the continuous flow reactor, Dalton et al. showed that TiO_2_ was effective in converting NO_2_ to non-harmful nitrate species under UV radiation [15]. However, the release of NO_x_ at higher temperatures has been found to be a critical problem when TiO_2_ is used in NO_x_ storage technology [16]. More efforts are still needed to obtain fundamental insights into the NO_2_ adsorption mechanism and to design more sensitive gas sensing devices.

Due to the superior surface-to-volume ratios and massive reaction sites, two-dimensional (2D) materials recently have been receiving attention for gas adsorption, including layered group III-VI semiconductors [17,18], h-BN [19,20,21], phosphorene [22], transition-metal chalcogenides (TMDs) [23,24,25], and so on. Lately, a new 2D material, namely Janus 2D TMD material, referring to layers with different surfaces, has generated urgent research interest in energy conversion applications [26,27,28,29]. There is a promise that the intrinsic dipole caused by the out-of-plane asymmetric structure in Janus 2D TMD materials tunes the adsorption of molecules on the surface, similar to the situation where an external vertical electric field markedly regulates the gas selectivity and sensitivity of MoS_2_ [30]. As a typical Janus 2D TMD material, the Janus WSSe monolayer has been successfully fabricated by implanting Se species into a WS_2_ monolayer with pulsed laser ablation plasmas [31] and by heating mixed WS_2_ and WSe_2_ powders at 1000 °C [32]. Although the adsorption performance on NO_2_ gas for WSe_2_, the parent material, has been investigated [33], the performance of the Janus WSSe monolayer remains unclear.

In this work, we have explored the adsorption of NO_2_ over pristine and Se-vacancy defect-engineered WSSe monolayer with DFT calculations. A systematical discussion of adsorption energy, density of states (DOS), and charge density difference (CDD) is presented to interpret the interaction between the NO_2_ gas and the substrate. We found that introducing Se vacancy could cause a physical-to-chemical adsorption transition for NO_2_ gas on the Janus WSSe monolayer. Keeping the S-rich environment and applying compression strain are two potential approaches for introducing Se vacancies into the Janus WSSe monolayer. Moreover, for sensing applications, it is also necessary to take into consideration of the desorption behavior of the gas, which could be characterized by the recovery time [34]. Usually, an intense binding suggests that desorption of gas molecules may be challenging, and the device may experience longer recovery times. Based on the Van’t Hoff–Arrhenius theory, the recovery time for NO_2_ gas adsorption on the pristine and defective Janus WSSe monolayer are investigated, respectively. The purpose of this work is to gain a fundamental understanding of the adsorption of NO_2_ gas on the Janus WSSe monolayer and how to design more sensitive gas treatment devices.

## 2. Results and Discussion

### 2.1. The Physisorption of NO_2_ on Pristine Janus WSSe Monolayer

#### 2.1.1. Screening of Adsorption Sites and Adsorption Energy

**Figure 1:**
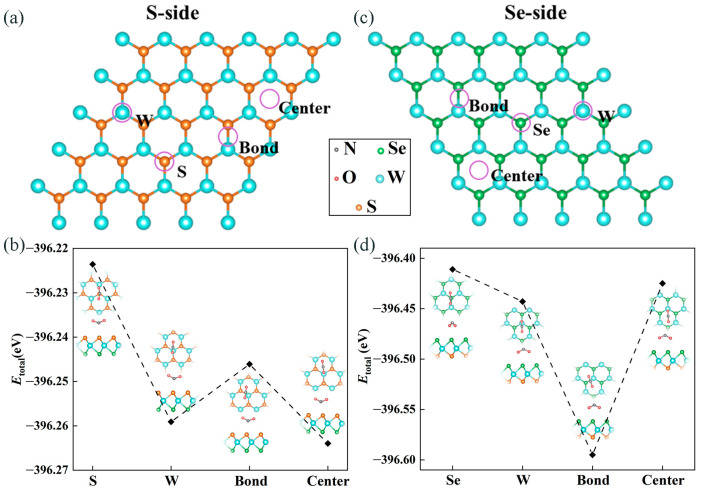
The purple circles indicate the adsorption sites considered in our work at the (**a**) S and (**c**) Se side of the pristine Janus WSSe monolayer. The total energy of a NO_2_ gas molecule adsorbed pristine WSSe monolayer with the four adsorption sites on the (**b**) S and (**d**) Se sides, respectively. The illustrations present the top (**upper**) and side (**lower**) views of the optimized configurations of these adsorption systems. The gray, red, orange, green, and blue balls represent N, O, S, Se, and W atoms, respectively.

The Janus WSSe single layer is formed by the W layer sandwiched between S and Se layers. It has a honeycomb structure similar to the one of its parent materials (WSe_2_ and WS_2_) [35]. The lattice constant of Janus WSSe is calculated to be 3.26 Å, which sits between the values of its parent materials (WSe_2_ and WS_2_). Similar to the case of Janus MoSSe [36], the out-of-plane intrinsic dipole, caused by structural asymmetry in the Janus WSSe could be expected to improve the gas sensing properties, which is highly desirable to explore. As shown in Figure 1a,c, in this work, we initially considered the adsorption geometries of NO_2_ on both sides of Janus WSSe. For each adsorption case, a gas molecule was placed on the top of a 4×4 supercell of the WSSe monolayer, and the whole system was fully relaxed. Furthermore, several possible adsorption sites were considered, including the top site above the center of the hexagon (denoted as **Center**), the top of the W/Se/S atom (denoted as **W/Se/S**), and the top site above the W-Se/(W-S) bond (denoted as **Bond**), with the configurations of the molecule being parallel to the monolayer surface.

According to Equation (1), it could be found that the $E_{\mathrm{ads}}$ was dominated by $E_{\mathrm{total}}$ because the $E_{\mathrm{sub}\;}$ and $E_{\mathrm{gas}}$ were constant at different adsorption sites. Here, the total energy of the gas molecules adsorbed on the Se and S sides of the Janus WSSe at different adsorption sites was calculated to explore the most stable adsorption configuration. As displayed in Figure 1b, for the adsorption of NO_2_ on the S-side, we found that the total energy reached a minimum (−396.26 eV) when it was located above the center of the hexagon (**Center** site), suggesting the most stable adsorption configuration. As to the case of the NO_2_ gas molecule adsorbed on the Se-layer, as illustrated in Figure 1d, it was discovered that, when the molecule was located on the top of the W-Se bond (**Bond** site), the system had the lowest total energy (−396.59 eV), which means it was the most stable adsorption site. Moreover, because the total energy of the most stable adsorption configuration on Se-side was 0.33 eV lower than the one on S-side, it could be obtained that the NO_2_ gas molecule preferred to adsorb on the Se-side. Hence, for the case of the NO_2_ gas molecule adsorbed on the pristine Janus WSSe, we focused on the adsorption configuration, with NO_2_ on the **Bond** site at the Se-side. The adsorption energy ($E_{\mathrm{ads}}$) of this adsorption configuration was −0.56 eV, indicating that this adsorption likely belonged to physisorption, where the absolute value of $E_{\mathrm{ads}}$ is normally less than 1 eV [34,37,38,39]. Further exploration of the physisorption is discussed in the following section.

#### 2.1.2. Adsorption Mechanism

The work mechanism for this physisorption of NO_2_ gas molecule adsorbed on pristine WSSe monolayer was meticulously investigated from the aspect of adsorption distance, CDD, Bader charge analysis, and DOS.

**Figure 2:**
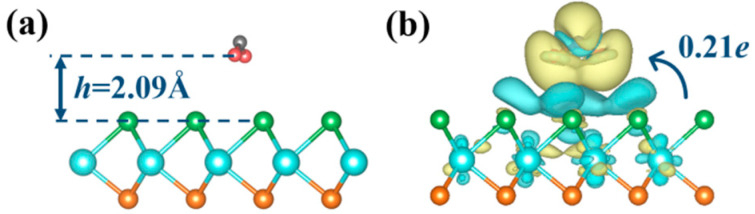
The side view of optimized structure (**a**) and the charge density difference (**b**) for the pristine Janus WSSe monolayer with NO_2_ gas molecule adsorbed on it. The adsorption distance between the gas molecule and substrate is denoted by *h* in dark blue. Areas in yellow (cyan) denote charge accumulation (depletion). The isosurface value is set to 0.0003 e Å^−3^. The charge transfer between the molecule and the substrate is denoted.

As plotted in Figure 2a, after adsorption, the oxygen atoms of the NO_2_ gas molecule tended toward the monolayer, and the NO_2_ gas molecule remained parallel with the monolayer with a vertical distances of 2.09 Å away from the pristine Janus WSSe monolayer. Moreover, the shortest distances between the O atom from NO_2_ molecule and its nearest Se atom was as large as 2.72 Å, greatly beyond the length of Se-O bond (1.81 Å). In addition, as seen in Figure 2b, there were merely 0.21 electrons transferring from the pristine Janus WSSe monolayer to the NO_2_ gas molecule, showing the feeble interaction that existed between the substrate and the gas molecule.

**Figure 3:**
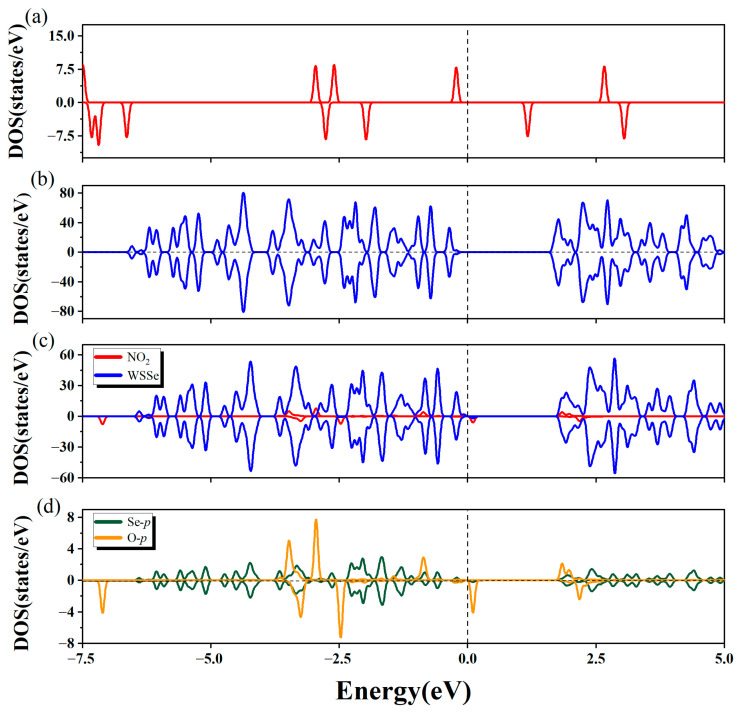
The total density of states of isolated NO_2_ gas molecule (**a**) and clean pristine WSSe monolayer (**b**). (**c**) The partial density of states of the adsorption system. WSSe portion is denoted in dark blue, while NO_2_ portion is denoted in red. (**d**) The partial density of states of O *p* orbitals (denoted in orange) from adsorbed NO_2_ gas molecule and Se *p* orbitals (denoted in green) from the Se atoms in the substrate, which is closest to the gas molecule. The vertical dashed line indicates the Fermi level.

The relevant DOS of this adsorption configuration was calculated. As illustrated in Figure 3a–c, both monolayer and gas molecule hardly changed after adsorption in terms of DOS, which was in accordance with the tiny interface transfer electron, suggesting the electronic property of WSSe and NO_2_ had no evident changes. There was little orbital hybridization between WSSe monolayer and NO_2_, which demonstrated that the interaction between the monolayer and molecule was poor, conforming to the discussion above. The orbital hybridization focused mainly on the energy intervals of −3.76~−3.10 eV and 1.71~2.10 eV, dominantly contributed by the Se *p* orbital from the WSSe monolayer and the O *p* orbital from the NO_2_ gas molecule (seeing Figure 3d). In addition, the weak orbital hybridization slightly delocalized the DOS peaks of the NO_2_ gas molecule, causing its integral area to increase gently. This agreed well with the small amount of gained electron (0.21 *e*) from the WSSe monolayer. According to the above analysis, the adsorption of NO_2_ on the pristine WSSe could be confirmed to be physisorption.

### 2.2. The Chemisorption of NO_2_ on Defective Janus WSSe Monolayer

The adsorption of NO_2_ gas molecule on the pristine WSSe is physisorption, which could be utilized as gas collection system. However, for the purpose of treating gas or speeding up chemical reaction, the chemisorption of NO_2_ was more essential, which necessitated a more powerful adsorption capacity of the substrate. On the basis of the previously relevant results, introducing some vacancy defects was found to have the ability to influence the electronic property and then improve the stability of some geometric structures effectively [40,41]. Thereby, we introduced vacancy defects in the Janus WSSe monolayer, hoping to obtain an enhanced adsorption capacity of NO_2_ gas molecule.

#### 2.2.1. Vacancy Screening

As shown in Supplementary Materials Figure S1, there are two types of vacancy defects in the monolayer WSSe considered, namely sulfur vacancy defect (S vacancy) and selenium vacancy defect (Se vacancy). The formation energies of the defective WSSe monolayer were calculated and are presented in Table 1. It can be seen that the S vacancy had a considerable positive formation energy under both S-rich and Se-rich conditions; the Se vacancy possessed a positive formation energy under the Se-rich condition as well, indicating that the structures of defective WSSe with these vacancies under the corresponding environments were unlikely to form. However, the formation energy of Se vacancy in the S-rich condition was negative, which means that the Se-vacancy could more easily generate Janus WSSe in an S-rich environment. Therefore, in the following calculation, the Se vacancy defect was highly appreciated and was adopted to improve the adsorption capacity of NO_2_ on the Janus WSSe monolayer.

#### 2.2.2. Screening of Adsorption Sites and Adsorption Energy

**Figure 4:**
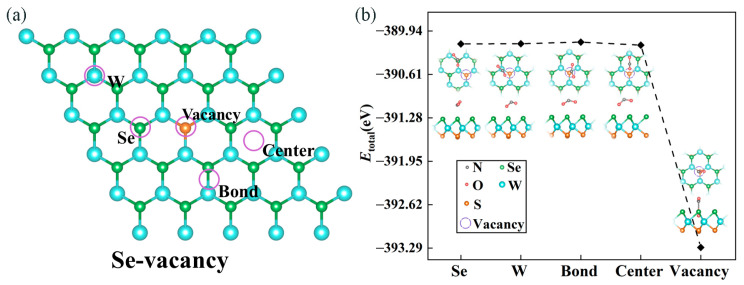
(**a**) The purple circles indicate the adsorption sites, considered in our work, at the defective Janus WSSe monolayer. (**b**) The total energy of a NO_2_ gas molecule adsorbed defective WSSe monolayer with the five adsorption sites. The illustrations present top (**upper**) and side (**lower**) views of the optimized configurations of these systems. The gray, red, orange, green, and blue balls represent N, O, S, Se, and W atoms, respectively.

As plotted in Figure 4a, five possible adsorption sites in the defective WSSe monolayer were considered, i.e., the top site above the center of the hexagon (denoted as **Center**), the tops of the W and Se atoms (denoted as **W** and **Se**, respectively), the top site above the W-Se bond (denoted as **Bond**), and the Se vacancy defect (denoted as **Vacancy**). Similar to the case of NO_2_ adsorbing on the pristine WSSe monolayer, we employed the total energy of the adsorption system to grasp the most stable adsorption configuration. As displayed in Figure 4b, the total energy attained a minimum when NO_2_ adsorbed on the **Vacancy** site, indicating that this site was the most stable location for NO_2_ adsorbing on defective WSSe monolayer. In this case, the $E_{\mathrm{ads}}$ value was −3.53 eV, which was approximately an order of magnitude more negative than that for NO_2_ adsorbing on the pristine WSSe monolayer (seeing Table S1). Apparently, the introduction of Se vacancy would effectively make the NO_2_ adsorb more strongly on Janus WSSe. On the basis of the exceptionally negative $E_{\mathrm{ads}}$, we could preliminarily judge that this adsorption behavior belonged to chemisorption, which is further addressed hereinafter.

#### 2.2.3. Adsorption Mechanism

To pursue a more in-depth understanding of NO_2_ adsorption on the defective Janus WSSe monolayer, we investigated the adsorption system in terms of the N-O bond length, CDD, electron transfer, and DOS.

**Figure 5:**
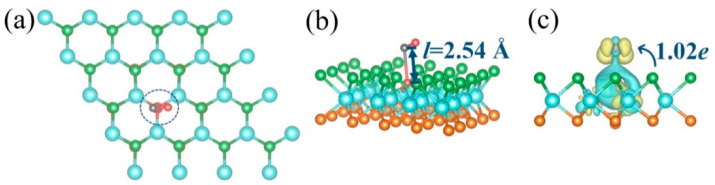
The top (**a**) and side (**b**) views of the optimized structures, as well as the CDD (**c**) of the defective Janus WSSe monolayer with NO_2_ gas molecules adsorbed on it. The N-O bond length is denoted by *l* in dark blue. Areas in yellow (cyan) denote charge accumulation (depletion). The isosurface value is set to 0.002 e Å^−3^. The charge transfer between the molecule and the substrate is denoted.

As shown in Figure 5a, one of the N-O bonds adopted nearly vertical orientation, with the oxygen atom pointing at the monolayer surface. Additionally, the oxygen atom formed bonds with the three adjoining tungsten atoms at the monolayer surface. Hence, the adsorption behavior definitely was chemisorption, which is in accordance with the result brought by its adsorption energy mentioned above. In addition, in order to quantitatively analyze the behavior changes of gas molecules before and after adsorption, we also measured the length of N-O bonds. Therein, we found that all the N-O bond lengths were 1.20 Å before adsorption, but one of the N-O bond length stretched to 2.55 Å after adsorption (seeing Figure 5b), denoting that the electrons in the gas molecule NO_2_ rebuilt after adsorption. As seen in the Figure 5c, there were significant charge redistributions in the adsorption system, and quite a few electrons (1.02 *e*) migrating from the defective Janus WSSe layer to the adsorbate. Normally, adsorption-induced charge transfer can cause resistivity variation of the system, which is an important index to show the sensing merit and can be measured experimentally for gas sensors [42,43].

**Figure 6:**
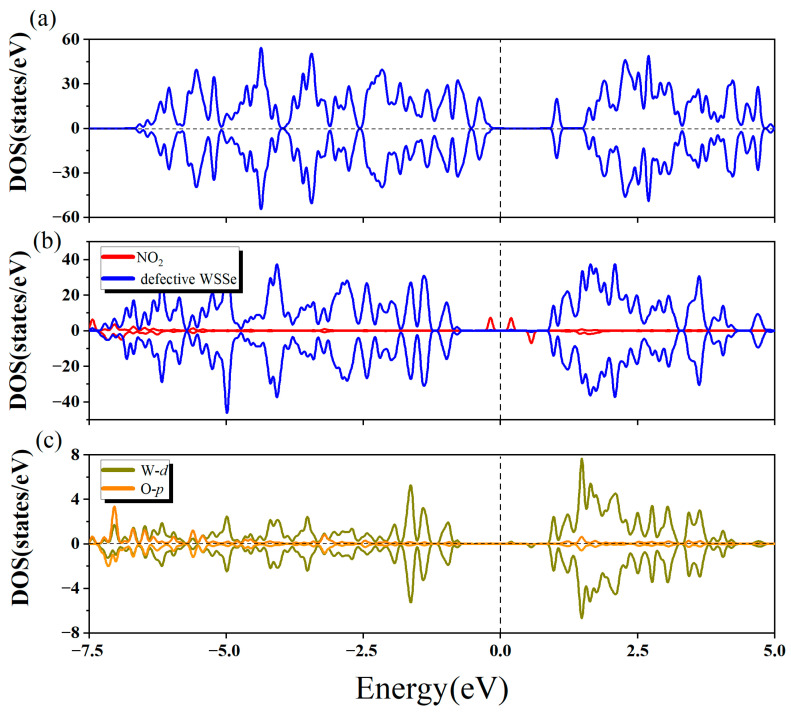
(**a**) The total density of states of clean defective Janus WSSe monolayer. (**b**) The partial density of states of the adsorption system. WSSe portion is denoted in dark blue, while NO_2_ portion is denoted in red. (**c**) The partial density of states of O *p* orbitals (denoted in orange) from adsorbed NO_2_ gas molecule and W *d* orbitals (denoted in dark yellow) from the three W atoms in the substrate, which bond to the O atom from the gas molecule. The vertical dashed line indicates the Fermi level.

To gain further insight into the electronic properties of the chemisorption system, we computed the relevant DOS and present them in Figure 6. A significant hybridization existed between the NO_2_ gas molecule and the defective Janus WSSe monolayer, which largely concentrated between −2.5 eV~−7.5 eV (seeing Figure 6b). This revealed that there was a strong interaction between them, explaining the phenomenon that NO_2_ was tightly attached to the defective WSSe monolayer. Furthermore, as shown in Figure 6c, the interaction was contributed mainly by the hybridization between O *p* orbital from the NO_2_ gas molecule and the W *d* orbital from the W atoms in the defective WSSe, which bonded to the O atom from the NO_2_ gas molecule. By comparing the DOS of NO_2_ gas molecule before and after adsorption (seeing Figure 3a and Figure 6b), it could be discovered that the DOS became delocalized significantly after adsorption, implying that the dramatic redistribution of electrons appeared in the NO_2_ gas molecule, which was the reason for the visible N-O bond alteration. Moreover, as shown in Figure 6a,b, the position of the valence band maximum (VBM) of the defective Janus WSSe monolayer moved downward after the NO_2_ gas molecule adsorbed on it. The drop in the VBM position corresponded with the Bader charge results, which demonstrated that the defective Janus WSSe monolayer lost 1.02 *e*. These outcomes further proved that the adsorption of NO_2_ on the defective WSSe monolayer belonged to chemisorption. That is to say, on Janus WSSe, introducing Se vacancy could wonderfully convert the physisorption of NO_2_ into chemisorption.

Additionally, though the above calculation results suggest that the defective Janus WSSe monolayer exhibits much improved sensing properties than the pristine one, it is worth noting that the stronger binding may also cause the desorption of the NO_2_ gas molecules from the defective Janus WSSe monolayer to be more difficult, and the devices may suffer from longer recovery times. In particular, for the defective Janus WSSe monolayer, the calculated recovery time (10^46^ s) was 10^50^ times that for pristine Janus WSSe monolayer (10^−4^ s) at room-temperature (300 K). Therefore, common methods, for instance, annealing in a vacuum and short UV irradiation [20], likely were not able to regenerate the defective Janus WSSe monolayer to its initial state. However, on the basis of the observation of N-O bond elongation in NO_2_ after adsorption, we suppose that NO_2_ reduction reaction (NO_2_→NO_2_^-^) is likely to take place [44], allowing the defective Janus WSSe monolayer to be reversible through water washing, which requires subsequent further investigations.

### 2.3. Compression Strain Facilitates Vacancy Formation

#### 2.3.1. Strain-Dependent Formation Energy

As is stated above, the S-rich environment is conductive to the formation of Se vacancy in the Janus WSSe monolayer. For a more effective introduction of vacancy in Janus WSSe, some other active methods would still be worth exploring. It is well known that strain can dramatically change the spatial structure and electronic properties of 2D materials [26,45,46,47,48]. Therefore, we explored how the strain effected the formation energy of Se vacancy in Janus WSSe, aiming to lower the formation energy with appropriate strain.

**Figure 7:**
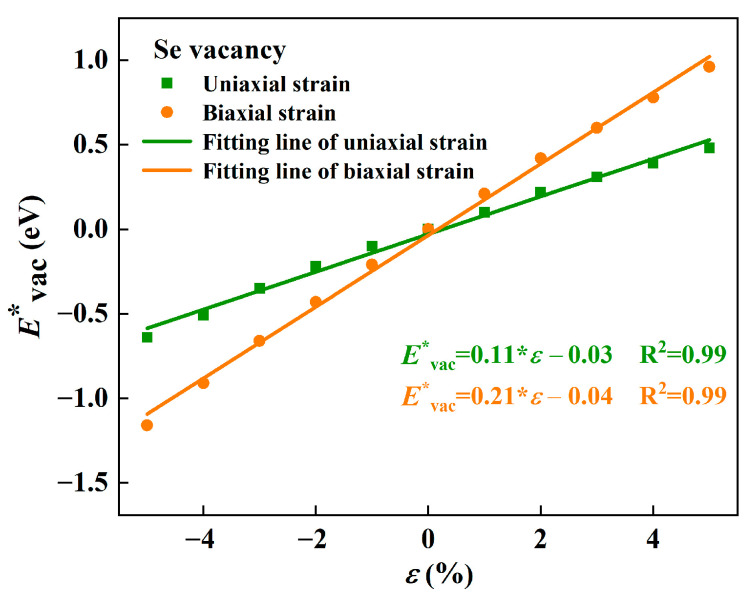
The relative $E_{\mathrm{vac}}^{*}\;$of Se vacancy under the different uniaxial (green) and biaxial strains (orange). The value of $E_{\mathrm{vac}}^{*}$ under no strain is selected as a reference value.

As plotted in Figure 7, there was a linear relationship between $E_{\mathrm{vac}}^{*}$ and *ε*, whether under the uniaxial or the biaxial strains. Furthermore, the greater the compression (smaller the tensile) strains exerted were, the lower the $E_{\mathrm{vac}}^{*}$ became, indicating that the formation energy of Se vacancy decreased linearly as the compression strain rose (tensile strain reduced). That is to say, Se vacancy can be formed more easily under compression strains, which provides a favorable way to generate Se vacancy.

In order to give a comprehensive picture of the influence on the formation of vacancy brought by the strain, we also tested the strain effect on S vacancy in the Janus WSSe monolayer. As presented in Figure S2, interestingly, the strain effect on S vacancy was similar to that on Se vacancy, which also had a linear relationship between$\;E_{\mathrm{vac}}^{*}$ and ε. Specifically, the compression strain induced a drop of the formation energy, while the tensile strain caused the formation energy to increase. Therefore, applying the compression stress can make it easier to form for both S and Se vacancies in the Janus WSSe monolayer. Furthermore, we supposed that it may be an effective method to generate vacancy for other similar structures.

#### 2.3.2. Origin of the Strain-Dependent Vacancy Formation

To analyze the underlying physical mechanism of the strain-dependent behavior of vacancy formation, we calculated the charge difference of the pristine WSSe with and without strain by employing Bader charge analysis. Considering that the influence taken by the −5%~5% strains were not obvious enough, here, we used 10% strain to enlarge the effect.

We calculated the charge of Se atom under −10%, 0, and 10% strain, respectively. As shown in Table S2, the valence electron of Se atom was reduced from 6.46 *e* to 6.41 *e* when the exerted strain dropped from 10% to −10%. Compared with the valence electron of Se atom under no strain, the one under −10% strain was closer to 6 *e*, which was the valence electron of isolated elemental selenium. This demonstrated that the gain electron of Se atom from W atoms became fewer under −10% strain, and then the interaction between the Se atom and its surrounding W atoms weakened. Therefore, the Se atom would be more likely to escape from the Janus WSSe monolayer, forming Se vacancy. As to the case of 10% strain, the valence electron of Se atom was greater than the one without strains. This suggested that, the electron transfer increased and the interaction between the Se atom and its adjoining W atoms was enhanced, making the separation of Se atoms from the Janus monolayer more difficult.

### 2.4. Physical-to-Chemical Adsorption Transition

**Figure 8:**
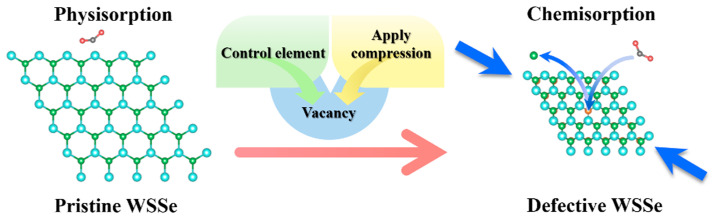
The schematic diagram of NO_2_ physical-to-chemical adsorption transition on Janus WSSe monolayer caused by the introduction of Se vacancy. The blue arrows at the opposite corners represent the direction of the imposed compression strain.

Based on the discussion above, the pristine Janus WSSe monolayer had a good physical adsorption capability to the NO_2_ gas molecule, which could be used to construct gas gathering system. By controlling stoichiometric proportions or applying compression strain, the vacancy, hopefully, could be introduced into the Janus WSSe monolayer. A physical-to-chemical adsorption transition was then caused by the vacancy, as displayed in Figure 8. The defective Janus WSSe monolayer exhibited a well chemisorption to the NO_2_ gas molecules, which could be applied to form exhaust gas processor components and gas detectors.

## 3. Conclusions

Owing to the potential environmental threats and commercial value of NO_2_ gas, the detection, collection, and handling of NO_2_ gas are considered critically necessary. In this work, we performed a theoretical study on the adsorption of NO_2_ on the pristine and defective WSSe monolayer. On the pristine WSSe monolayer, according to the tiny adsorption energy, long adsorption distance, and weak electronic orbital hybridization, the adsorption of NO_2_ gas molecule is verified to be physisorption. After adsorption, the electronic properties of NO_2_ gas molecule and the pristine Janus WSSe monolayer both are essentially the same as those in their isolated states. The introduction of Se vacancy in Janus WSSe monolayer, which could be promisingly realized by S-rich environment or applying compression strain, dramatically raises the transferred-electron quantities at the interface and induces an obviously electronic orbital hybridization between the adsorbate and substrate, causing the adsorption of NO_2_ gas molecule on the defective Janus WSSe monolayer to be chemisorption. The physical-to-chemical adsorption transition caused by the introduction of Se vacancy allows Janus WSSe monolayers to satisfy the different demands of different gas sensitive installations. The physisorption of NO_2_ gas molecule combined with the short recovery time makes the pristine Janus WSSe monolayer suitable for collecting and storing gases at low temperatures. Meanwhile, the powerful chemisorption of NO_2_ gas molecule affords defective Janus WSSe monolayers the potential to activate and reduce NO_2_ used for NO_2_ gas conversion. Our studies opens a new path for the adsorption of NO_2_ and provide a strong foundation for the development of the application of the Janus WSSe monolayer.

## 4. Computational Methods

In this study, the DFT calculations for the geometrical relaxation and electronic structure were carried out by using the Vienna Ab initio Simulation Package (VASP) (version 5.3, Hanger Group, University of Vienna) [49,50]. The generalized gradient approximation (GGA) method with Perdew–Burke–Ernzerhof (PBE) for the exchange–correlation energy was used. In order to describe the van der Waals (vdW) interaction between gas molecules and the substrate, we adopted the zero-damped DFT-D2 method proposed by Grimme [51]. The cutoff energy for the plane wave basis set was taken as 500 eV. During the optimization, all the internal coordinates were allowed to relax with a fixed lattice constant. Spin polarization was employed in the calculations of the adsorption of NO_2_ since the molecule is paramagnetic [52]. A 4 × 4 supercell of pristine or defective WSSe monolayer, with a single gas molecule adsorbed on it, was chosen as the computational model. Brillouin zone was sampled for integration according to Monkhorst–Pack scheme [53] with a 2 × 2 × 1 K point sampling for thermodynamic stability and electronic properties calculations. A vacuum of 30 Å was provided along c-direction to avoid the effect of interlayer interaction. It has been shown that the DFT method is considered to be one of the most accurate methods for calculating the electronic structure of solids [54,55,56].

The adsorption energy ($E_{\mathrm{ads}}$) of the NO_2_ on the pristine and defective WSSe monolayer was calculated from [57,58] (Equation (1)),

(1) $$E_{\mathrm{ads}}=E_{\mathrm{total}}-E_{\mathrm{sub}\;}-E_{\mathrm{gas}}\;$$

where$\;E_{\mathrm{total}}$ is the total energy of the gas-adsorbed monolayer, and $E_{\mathrm{sub}}$ and $E_{\mathrm{gas}}$ are the energies of the clean substrate (pristine or defective Janus WSSe monolayer) and the isolated NO_2_ gas molecule, respectively. A negative value of $E_{\mathrm{ads}}\;$indicates an exothermic adsorption. The more negative the $E_{\mathrm{ads}}$ is, the stronger the gas adsorption is.

The formation energy of defect *x* is defined by the following equation (Equation (2)),

(2) $$E_{\mathrm{vac}}^{f}\left(x\right)=E_{\mathrm{def}}\left(x\right)-E_{\mathrm{per}}-\mu_{i}$$

where $E_{\mathrm{def}}\left(x\right)$ is the total energy of a system containing an *x* defect, $E_{\mathrm{per}}$ represents the energy of a perfect supercell, and $\mu_{i}$ is the energy of *x* atom. The value of $\mu_{i}$ largely depends on the experimental growth conditions. For the Janus WSSe monolayer, on the basis of the previous fabrication process [31,32], we considered the S-rich and Se-rich conditions as the limiting cases to discuss the $E_{\mathrm{vac}}^{f}\left(x\right)$. In the thermodynamic equilibrium situation, one can assume that (Equation (3)),

(3) $$\mu_{\mathrm{wsse}}=\mu_{w}+\mu_{s}+\mu_{\mathrm{se}}$$

where $\mu_{\mathrm{wsse}}$ is the total energy per WSSe formula unit. Under the S-rich environment, the S chemical potential ($\mu_{s}^{0}$) is equal to the total energy per S atom in the S_2_ molecule. Then, the Se chemical potential can be written as (Equation (4)),

(4) $$\mu_{\mathrm{se}\left(S-\mathrm{rich}\right)}=\mu_{\mathrm{wsse}}-\mu_{w}^{0}-\mu_{s}^{0}$$

where $\mu_{w}^{0}$ is the total energy per W atom in its stable bulk phase. Meanwhile, for the Se-rich condition, the Se chemical potential ($\mu_{\mathrm{se}}^{0}$) is equal to the total energy per Se atom in its reference phase, i.e., the Se bulk having body-centered-cubic structure. The S chemical potential can be written as (Equation (5)),

(5) $$\mu_{s\left(\mathrm{Se}-\mathrm{rich}\right)}=\mu_{\mathrm{wsse}}-\mu_{w}^{0}-\mu_{\mathrm{se}}^{0}$$

The plane-integrated CDD was performed according to the following equation (Equation (6)),

(6) $$\Delta \rho =\rho_{\mathrm{total}}-\rho_{\mathrm{sub}}-\rho_{\mathrm{gas}}$$

where $\rho_{\mathrm{total}}$, $\rho_{\mathrm{sub}}$, and $\rho_{\mathrm{gas}},$ respectively, are the charge density of the gas-adsorbed system, substrate, and NO_2_ molecule.

From the Van’t Hoff–Arrhenius theory, the recovery time, τ, can be estimated by [34,59] (Equation (7)):

(7) $$\tau =\omega^{-1}\exp\left(\frac{E^{*}}{K_{B}T}\right)$$

where *T*, K_B_, E^*^, and *ω* stand for the temperature, Boltzmann Constant, desorption energy barrier, and attempt frequency, respectively. Here, E^*^ is approximated as the adsorption energy, while *ω* is assumed to be 10^13^ s^−1^ [34].

The strain is defined as (Equation (8))

(8) $$\varepsilon =\left(a\;–\;a_{0}\right)/a_{0}$$

where $a_{0}$ and a are the lattice parameters of the unit cell without and with strain, respectively. In this work, −5%~5% strain was considered, where the positive values mean tensile strains, while the negative values stand for compression strain.

The formation energy of the vacancy under the strain could be similarly defined by Equation (2), where $E_{\mathrm{def}}\left(x\right)$ and $E_{\mathrm{per}}$ are the corresponding values under the same strain, respectively. $\mu_{i}$, which is related only to the synthetic environment, has nothing to do with the exerted strain. Here, we define a new concept, $E_{\mathrm{vac}}^{*}$, as follows (Equation (9)):

(9) $$E_{\mathrm{vac}}^{*}\left(x\right)=E_{\mathrm{def}}\left(x\right)-E_{\mathrm{per}}$$

Since the $\mu_{i}$ is constant with different strain, the effect of strain was identical for both $E_{\mathrm{vac}}^{*}\left(x\right)$ and $E_{\mathrm{vac}}^{f}\left(x\right)$. Therefore, in the following, we substituted $E_{\mathrm{vac}}^{*}$ for $E_{\mathrm{vac}}^{f}$ to study the strain effect for convenience. Moreover, the $E_{\mathrm{vac}}^{*}$ under no strain was chosen as a criterion. In this case, the positive relative $E_{\mathrm{vac}}^{*}$ implied the increase of the formation energy, and the negative relative $E_{\mathrm{vac}}^{*}$ indicated the decrease. 

## Figures and Tables

**Table 1:** The formation energy of Se vacancy and S vacancy in the Janus WSSe monolayer under different synthetic conditions.

Vacancy	Synthetic Environment	
	S-Rich	Se-Rich
Se	−0.25 eV	2.78 eV
S	3.35 eV	0.32 eV

## Data Availability

The data presented in this study are available in Supplementary Materials.

## Supplementary Materials

The following supporting information can be downloaded at: https://www.mdpi.com/article/10.3390/molecules28041644/s1, Figure S1. The location of Se (a) and S vacancy defects (b) considered in our study; Figure S2. The relative $E_{\mathrm{vac}}^{*}$ of S vacancy under the different uniaxial (blue) and biaxial strains (orange); Table S1. The adsorption energy of NO_2_ gas molecules on pristine and defective Janus WSSe monolayer; Table S2. The calculated charge of one Se atom from Janus WSSe monolayer under −10%, 0 and 10% strain.
