# Supplementary material for: NO2 Physical-to-Chemical Adsorption Transition on Janus WSSe Monolayers Realized by Defect Introduction
Source: Molecules. 2023 Feb 8;28(4):1644. doi: 10.3390/molecules28041644 (PMC9960547; doi:10.3390/molecules28041644)
Supplement: Supplementary file 1 [file molecules-28-01644-s001.zip › molecules-2167502-supplementary.pdf]

Supporting information

NO_2 physical-to-chemical adsorption transition on Janus WSSe monolayers realized by defect introduction

Lin Ju[1,*], Xiao Tang[2], Xiaoxi Li[1], Bodian Liu[1], Xiaoya Qiao[1], Zhi Wang[1], and Huabing Yin[3,*]

[1]School of Physics and Electric Engineering, Anyang Normal University, Anyang, 455000, China

[2]College of Science, Institute of Materials Physics and Chemistry, Nanjing Forestry University, Nanjing 210037, China

[3]Joint Center for Theoretical Physics, and Institute for Computational Materials Science, School of Physics and Electronics, Henan University, Kaifeng 475004, China.

Correspondence: julin@aynu.edu.cn (L.J.); yhb@henu.edu.cn (H.Y.)

KEYWORDS: *gas sensing, WSSe monolayer, Se vacancy, density functional theory*

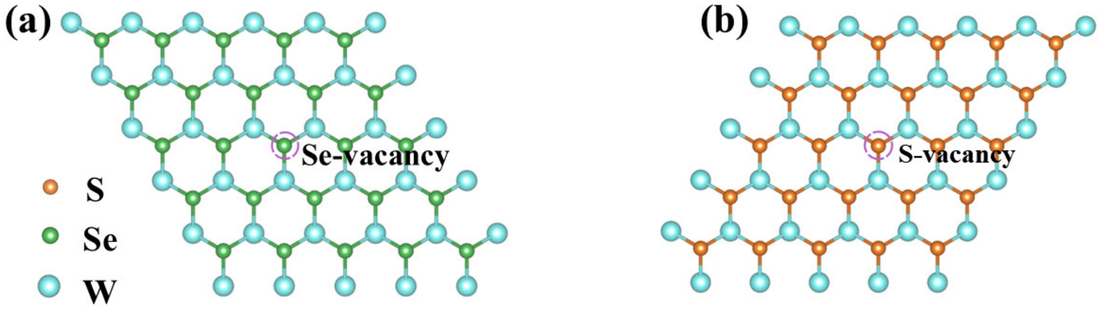

Figure S1. The location of Se (a) and S vacancy defects (b) considered in our study, which is circled by purple dashed line.

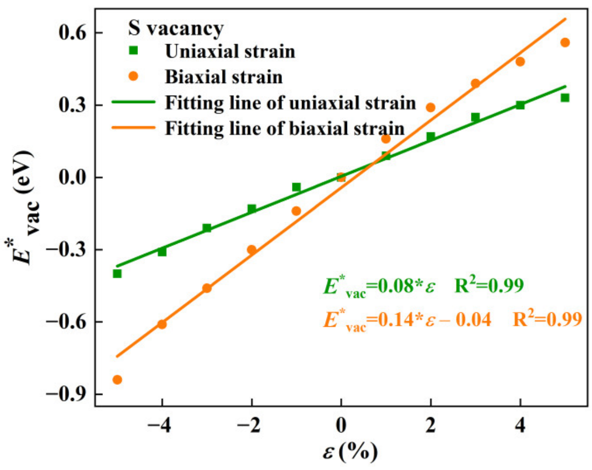

Figure S2. The relative E^*_{vac} of S vacancy under the different uniaxial (blue) and biaxial strains (orange). The value of E^*_{vac} under no strain is selected as a reference value.

Table S1. The adsorption energy of NO_2 gas molecules on pristine and defective Janus WSSe monolayer.

WSSe	Adsorption energy
Pristine	-0.56 eV
Defective	-3.53 eV

Table S2. The calculated charge of one Se atom from Janus WSSe monolayer under -10% , 0 and 10% strain.

Strain	Charge
-10%	6.41 *e*
0	6.44 *e*
10%	6.46 *e*
